# Human Milk Processing and Its Effect on Protein and Leptin Concentrations

**DOI:** 10.3390/nu15020347

**Published:** 2023-01-10

**Authors:** Christoph Binder, Sabina Baumgartner-Parzer, Liliana-Imi Gard, Angelika Berger, Alexandra Thajer

**Affiliations:** 1 Comprehensive Center for Pediatrics, Department of Pediatrics and Adolescent Medicine, Medical University of Vienna, 1090 Vienna, Austria; 2 Division of Endocrinology and Metabolism, Department of Internal Medicine III, Medical University of Vienna, 1090 Vienna, Austria

**Keywords:** pasteurization, freezing, thawing, human milk, protein, leptin, preterm, term, individualized target fortification, gestational age

## Abstract

(1) Background: For the storage of human milk (HM), freezing, thawing, and/or pasteurization are routinely used in neonatal intensive care units. We aimed to analyze the effects of different HM processing types on the nutritional contents in HM, adipose tissue, and the neuroprotection markers leptin and adiponectin. (2) Methods: HM samples from 136 mothers of preterm and term infants (gestational age 23 + 0 to 41 + 6) were collected and divided into four groups: (i) fresh HM, (ii) fresh pasteurized HM, (iii) thawed HM, and (iv) thawed pasteurized HM. The macronutrients were analyzed by mid-infrared transmission spectroscopy and the adiponectin and leptin were analyzed by high-sensitivity adiponectin and leptin enzyme-linked immunosorbent assay (ELISA). (3) Results: No significant differences were observed in the protein, carbohydrate, or fat concentrations between the HM processing types. The leptin levels were significantly lower after pasteurization in comparison to HM without pasteurization (*p* < 0.001). The protein levels in extremely preterm HM were significantly lower compared to those in moderate/late preterm HM and term HM (*p* < 0.05). (4) Conclusions: HM processing had an impact on leptin concentrations but no effect on the protein level. These data support the use of unpasteurized human milk for preterm infants’ nutrition and normal brain development. The protein levels of the milk of mothers from preterm compared to full-term infants differed, underlining the importance of individualized target fortification.

## 1. Introduction

Human milk (HM) is recommended for all infants and is the best source of nutrition for preterm and term infants. In particular, it is essential for preterm infants and supports the transition from intrauterine to extrauterine life. HM provides all the necessary nutrients the infant needs during the first months of life and encourages infants’ optimal growth and the development of organs and the immune system. The unique properties of HM, such as immunomodulatory, bioactive, and growth factors, digestive enzymes, hormones, prebiotics, and non-antibody mediated immune factors, enhance growth and metabolic maturation [1,2,3,4,5,6,7].

The macronutrient contents of HM are highly variable between mothers and maternal factors during pregnancy and after birth over the course of lactation, and these factors might affect the nutrient composition. HM contents are different from those in colostrum and differ between transitional and mature HM and between hindmilk and foremilk. Infant characteristics, such as birth weight, height, and length, gender of the infant, and gestational and postnatal age might play a role in the high variability of HM contents [3,8,9,10,11,12]. The comparison of preterm and term HM, and therefore, gestational age (GA), might be an important predictor of HM composition. However, previous studies have evaluated the composition of HM in preterm and term infants with inconsistent results. In a systematic review and meta-analysis, Gidrewicz and Fenton showed that gestational age was a major predictor of HM composition [8]. However, the impact of gestational age at birth on leptin and adiponectin concentrations has not yet been investigated and is a secondary aim of the current study. Detailed information on the composition of HM is of high interest in the care of preterm neonates because this would allow targeted nutritional management and individualized fortification concepts. These concepts in neonatal intensive care medicine are focused on optimizing nutritional management to improve long-term health outcomes in this vulnerable group of patients.

Studies have shown that the implementation of individualized target fortification is feasible and safe in clinical routines and has improved growth in preterm infants [13,14]. To implement individualized target fortification concepts in clinical routines, neonatologists need to know the daily nutritional contents of HM. The macronutrient contents, especially the protein levels, are substantial, as the requirements for preterm infants are higher compared to term infants [15]. Moreover, protein is the key nutrient for infant development, growth, and neurodevelopment [16,17,18]. In particular, in infants with extremely low birth weight, attention must be paid to their protein intake.

HM contains the hormones leptin and adiponectin. Adiponectin and leptin are primarily produced in the white adipose tissue, and serum leptin correlates with total body fat mass [19,20]. Adiponectin is highly concentrated in HM [21], and higher adiponectin levels in HM are associated with a higher fat mass [22]. The peptide hormone leptin regulates energy balance and suppresses food intake in adults [20,23]. Furthermore, it has been shown to play an important role in preterm infants’ growth and brain development [24,25]. In utero, high amounts of leptin are transferred from the placenta to the fetus in the last trimester [23]. Studies have demonstrated that the neurotrophic factor leptin dramatically decreases after preterm delivery [23,26]. Additionally, preterm infants have reduced leptin production, and, therefore, extremely preterm infants are at especially high risk for leptin deficiency [19,23]. Leptin receptors are widely distributed in the brain and affect brain development [26,27]. Several animal studies have demonstrated that leptin administration was associated with hypothalamic enlargement and improved neurodevelopment [24,25,28]. However, the mother’s own milk provides the optimum nutrition for preterm infants, and if it is not available, pasteurized donor HM is the best alternative. A previous study found that HM leptin concentrations were correlated with maternal serum concentrations [29]. An exploratory study with a small sample size showed that holder pasteurization had an impact on leptin concentrations in a combined study cohort with human breast milk and term donor HM samples [30]. However, the effect of HM processing (freezing, thawing, and/or pasteurization) on preterm mothers’ own breast milk alone has not thus far been investigated. The aim of the study was to investigate the effect of different human milk processing types on adiponectin and leptin concentrations in the mother’s own HM.

For the storage of HM samples, freezing, thawing, and/or pasteurization are routinely used in neonatal intensive care units. In neonatology, the processing of HM is widely used to reduce microbiological germs. Pasteurization eliminates microorganisms and decreases the protein, immunoglobulin A (IgA), lactoferrin, and energy concentrations in HM. Freezing decreases viral and bacterial activity. The freezing step and subsequent thawing decrease digestive enzymes such as lipases, amylases, and proteases [31,32].

The primary aim of our study was to analyze the effects of different processing types on the nutritional contents in HM, with a special focus on protein. The secondary aim was to investigate the effects of different HM processing types on leptin and adiponectin concentrations. Moreover, we aimed to determine the differences between preterm and term HM composition.

## 2. Materials and Methods

### 2.1. Recruitment

All mothers aged between 18 and 50 years who were breastfeeding or pumping for their preterm or term infant at the General Hospital of Vienna, Medical University of Vienna, Vienna, Austria were eligible for the study. Mothers with mastitis and mothers with insufficient milk production caused by medication, maternal illness (e.g., Sheehan’s syndrome, hypothyroidism, prolactinoma, and polycystic ovarian syndrome), maternal stress, and substance abuse were excluded. In addition, mothers of infants with gastrointestinal diseases, such as Hirschsprung disease, chronic inflammatory bowel disease, congenital heart disease, major congenital birth defects, and chromosomal aberrations were not recruited.

### 2.2. Study Design

In this prospective, monocentric study, we aimed to analyze the effects of HM processing on the HM composition of preterm and term lactating mothers. A total of 136 HM samples were collected from lactating mothers of preterm and term infants from March 2016 to July 2022. In total, 544 HM samples were analyzed. The human milk samples were divided into four groups according to the type of processing performed:

(i) Fresh HM;
(ii) Fresh pasteurized HM;
(iii) Thawed HM;
(iv) Thawed pasteurized HM.

**Figure 1:**
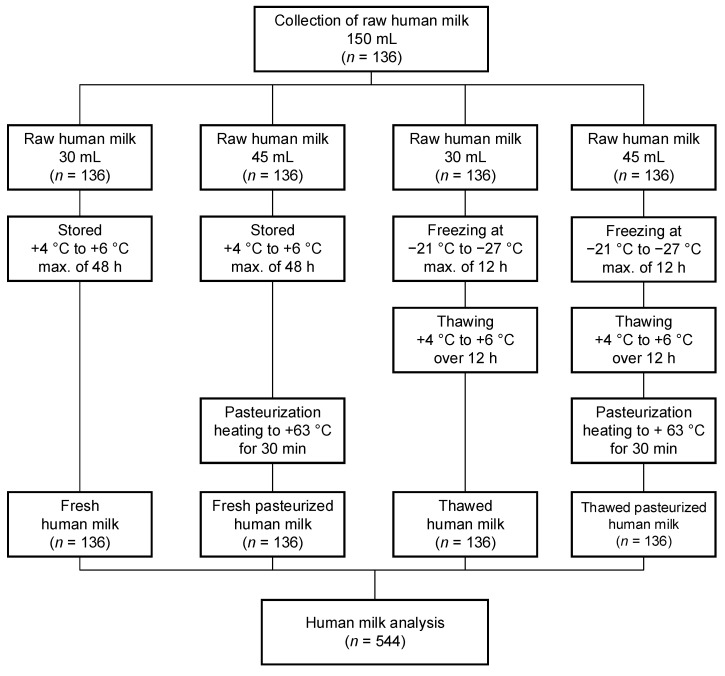
Description of study design. max., maximum.

The following diagram summarizes all study procedures within the project (Figure 1).

This study was approved by the local ethics committee (approval number 2022/2015, 05/FEB/2016) of the Medical University of Vienna. All lactating mothers gave their prior written informed consent.

### 2.3. Human Milk Collection

During this study, HM samples were collected in sterile feeding bottles from lactating mothers. Overall, an HM sample volume of approximately 150 mL was needed from each lactating mother. The volume of 150 mL was expressed within 24 h and was pooled into one sample per mother. The mothers expressed their HM samples directly in the ward in the hospital, and discharged mothers were advised to transport their HM samples in a cooling bag to ensure the cold chain continued at all times up to their transportation to the hospital. Each 150 mL HM sample was divided into four sterile bottles in the following volumes to determine the (i) fresh HM: 30 mL, (ii) fresh pasteurized HM: 45 mL, (iii) thawed HM: 30 mL, and (iv) thawed pasteurized HM: 45 mL (Figure 1). After the different processing types were carried out, only 3 mL was needed to conduct a measurement of an HM sample with the Miris Human Milk Analyzer^TM^ (Miris AB, Uppsala, Sweden). Duplicate measurements were carried out for each sample with this device; therefore, about 6 mL was needed. For the adiponectin and leptin measurements, only a volume of 1–2 mL HM was required. For both parameters, a double measurement was performed.

### 2.4. Human Milk Processing

The donated HM samples of each mother underwent the same processing steps in the milk kitchen at the Department of Pediatrics and Adolescent Medicine at the Medical University of Vienna. Each HM sample was divided into four groups according to the different processing types:

(i). Fresh HM: the expressed raw HM samples were stored at +4 to +6 degrees Celsius in the refrigerator for a maximum of 48 h;
(ii). Fresh pasteurized HM: the expressed raw HM samples were stored at +4 to +6 degrees Celsius in the refrigerator for a maximum of 48 h and were pasteurized by heating the samples to +63 degrees Celsius for thirty minutes with the Barkey clinitherm Pasteur XPT (Barkey GmbH & Co. KG, Leopoldshöhe, Germany);
(iii). Thawed HM: the expressed raw HM samples were frozen at −21 to −27 degrees Celsius for a maximum of 12 h, followed by thawing at +4 to +6 degrees Celsius in the refrigerator over twelve hours;
(iv). Thawed pasteurized HM: the expressed raw HM samples were frozen at −21 to −27 degrees Celsius for a maximum of 12 h, followed by thawing at +4 to +6 degrees Celsius in the refrigerator over 12 h, and additionally followed by pasteurization by heating the samples to +63 degrees Celsius for thirty minutes with the Barkey clinitherm Pasteur XPT (Barkey GmbH & Co. KG, Leopoldshöhe, Germany).

### 2.5. Human Milk Analysis

The Miris Ultrasonic Processor^TM^, the Miris Heater^TM^, and the Miris Human Milk Analyzer^TM^ (Miris AB, Uppsala, Sweden) were used for the HM analysis according to the manufacturer’s operating instructions (MIRIS AB, Uppsala, Sweden) [33,34,35]. The Miris Ultrasonic Processor^TM^ was utilized for the homogenization of the milk samples based on high-frequency ultrasonic waves [35]. The Miris Heater^TM^ is a water-free system and was applied to heat the milk samples to the optimum temperature for analyses [34]. The HM composition was evaluated with the Miris Human Milk Analyzer^TM^ (HMA). The HMA is an analytical instrument and is designed for the bedside analysis of HM based on a combination of mid-infrared (IR) transmission spectroscopy for the direct determination of the nutritional contents of HM [33]. The HM composition was calculated on the basis of spectral content. The HMA analyzed the true protein (g/100 mL), crude protein (g/100 mL), fat (g/100 mL), and carbohydrate (g/100 mL) levels in the HM samples. Further, the total solids (g/100 mL) and energy (kcal/100 mL) were calculated. For one measurement, only 3 mL was needed. The HM samples were measured twice, and the average value was taken.

The leptin and adiponectin concentrations were analyzed in the HM samples using the respective leptin and high-sensitivity adiponectin enzyme-linked immunosorbent assay (ELISA) kits purchased from BioVendor (Brno, Czech Republic). An application protocol was used, which was proven for leptin and adiponectin measurements in HM. Assay procedures were performed as recommended by the manufacturer, and the samples were analyzed in duplicate. For the leptin assay, the samples used were undiluted, whereas the adiponectin was analyzed in HM samples diluted 3× in dilution buffer [36,37,38].

### 2.6. Statistical Methods

The primary objective of the present study was to determine the protein contents of HM under different types of HM processing. In addition, the secondary objectives were related to the fat, carbohydrate, energy, total solids, adiponectin, and leptin contents of HM under different types of HM processing. The results of the primary and secondary objectives are expressed as the median and range or mean and standard deviation (SD) in the tables and the text. Given the non-normal distribution of the data, all comparisons were performed using nonparametric tests. The Kruskal-Wallis ANOVA was followed by a Mann-Whitney U-test for a comparison of the protein, fat, carbohydrate, energy, total solids, adiponectin, and leptin levels under different HM processing methods. The nutritional milk compositions of the four different processing types were compared by a Friedman test. The Mann-Whitney U-test was used to compare differences in the HM composition between the gestational age (GA) groups. Data analysis was carried out using the Statistical Package for Social Science (SPSS) for Windows (version 26.0, SPSS Inc., Chicago, IL, USA). A *p*-value of < 0.05 was considered statistically significant.

### 2.7. Sample Size Calculation

The primary question was whether there was a distinction between different processing groups of HM samples of preterm and term lactating mothers in the means of protein. A sample size of 136 in each group had 80% power to detect a difference in means of 0.13 assuming that the common standard deviation was 0.38 using a two-group t-test with a 0.050 two-sided significance level. Therefore, we aimed to enroll at least 136 lactating mothers.

## 3. Results

### 3.1. Participant Characteristics

A total of 136 lactating mothers were included in this study; 91% had a cesarean delivery, and a high percentage (93%) gave birth to a preterm infant. The maternal and infant characteristics are summarized in Table 1.

### 3.2. Human Milk Processing and Human Milk Composition

The HM compositions of the different processing types are reported in Table 2. The protein contents—true as well as crude protein—in the HM did not differ between the types of processing. All types of processing significantly differed in the leptin concentrations (*p* < 0.001). Pasteurization decreased the nutrient value in the HM in all measured parameters (n.s.) and was significantly different in regard to leptin (*p* < 0.001).

### 3.3. Human Milk Composition of Mothers from Preterm and Term Infants

The 136 mothers and their infants were assigned to four groups according to the infants’ gestational age (GA). Out of the 136 born infants, a total of 126 (93%) were preterm infants, and thereof, 34 (25%) infants were born moderate to late preterm (GA: 32 to <37 weeks), 30 (22%) were born very preterm (GA: 28 to <32 weeks), and 62 (46%) were born extremely preterm (GA: <28 weeks). Table 3 shows the characteristics of the mothers and their infants grouped according to the GA. 

Table 4 depicts the HM, which was divided into different groups according to the GA of the infants.

Statistically significant differences were found between the groups of preterm and term lactating mothers with respect to protein. Mothers with extremely preterm infants showed significantly lower protein contents (both true and crude protein) (*p* = 0.001), energy levels (*p* = 0.022), and total solids (*p* = 0.023) compared to mothers of moderate to late preterm infants. Moreover, extremely preterm HM had significantly lower true protein (*p* = 0.009) and crude protein (*p* = 0.012) levels compared to term HM. The nutritional HM contents were significantly lower in mothers with very preterm infants compared to moderate/late preterm infants in regard to true protein (*p* = 0.030), crude protein (*p* = 0.037), carbohydrates (*p* = 0.032), energy (*p* = 0.017), and total solids (*p* = 0.006).

## 4. Discussion

In the present study, we examined the impact of processing HM from the mothers of preterm and term infants. The main observations from the present study highlight that processing had no impact on the protein content or the other macronutrients, fat, and carbohydrates. However, contrary to the protein concentrations, the leptin levels in HM were affected by processing. HM composition is associated with the prematurity of the HM. The protein levels were significantly lower in HM from preterm infants in comparison to term infants.

The outcomes of HM processing are not homogenous or even contradictory. The HM composition might affect the quality and quantity of HM components through different methods of storage, freezing, thawing, pasteurization, and HM analysis [39,40,41,42].

Studies have shown that pasteurization via Maillard reaction and lipid oxidation leads to protein carbonylation on amino acid residues [43,44]. This might be the reason why Vieira et al. indicated a 4% protein reduction after HM pasteurization [45]. However, the results of the current study show that pasteurization had no impact on the HM protein content, which is in line with results previously published in the literature [46,47,48]. Valentine et al. analyzed the amino acid levels in HM and did not find significant differences before and after pasteurization [49]. Kotrri et al. measured the total amount of nitrogen in HM with elemental analysis, and it remained stable after pasteurization and the chemical structure did not change significantly [47]. Espinosa-Martos et al. observed that the lactose contents in colostrum did not change significantly after pasteurization [50]. Peila et al. found a non-significant total protein decrease after HM pasteurization [48]. It can be concluded that HM pasteurization preserves the biological activity of proteins and amino acids remain stable, and therefore, are available for an infant’s enteral intake. Collectively, our data show that all types of HM processing did not affect the protein concentrations, including true and crude protein. Therefore, the protein levels in HM seemed to be stable, and heating and freezing had no impact on this macronutrient. This is in line with other studies and represents a benefit in the nutritional management of preterm infants [51,52].

Our method used in the milk kitchen seemed to not accelerate the heat-induced denaturation and aggregation of whey proteins. However, the following factors, such as temperature, duration, and the method of pasteurization, might have had an impact on the findings [46]. Moreover, another study assessed that HM processing did not affect protein concentrations or any other HM nutrient but had an impact on osmolarity and should be especially considered in combination with fortification steps performed in preterm infants [51].

Freezing and thawing especially interact with the macronutrient fat contents due to the changes in fat globules [53,54,55]. With regard to pasteurization, evidence has been accumulating over the last few years on the decreasing effect on fat [56]. We found that fat contents were reduced after pasteurization, but the results are not significantly different, and these findings are in line with the literature [57]. Previous studies have revealed that pasteurization decreased the activity of immune and bioactive components, such as growth factors and adipokines [39,58]. Our study corresponds to these previous studies, and we observed that the heating process decreased the level of adiponectin in HM, and leptin seemed to be almost completely inactivated by pasteurization. Vass et al. [30] showed that pasteurization decreased leptin concentrations in a combined study cohort with human breast milk and term donor milk samples in a relatively small sample size (*n* = 55). In our study, different HM processing methods and their effect on leptin and adiponectin were investigated in the mothers’ own milk only. We demonstrated that pasteurization but not freezing alone had an impact on human breast milk in a large study cohort of 136 HM samples. To our knowledge, this is the largest study evaluating the effects of different milk processing methods on leptin and adiponectin levels in preterm human breast milk. Furthermore, we confirmed the hypothesis that pasteurization had a negative impact on leptin concentrations in human breast milk. Long-term follow-up studies, especially in preterm infants, are of major interest to evaluate whether this might translate into impaired neurodevelopment in these infants.

A plausible explanation for this finding could be that the functional activity of leptin is destroyed by temperature changes. The heating process, performed as pasteurization, decreased, even almost destroyed completely leptin levels. However, little is known about leptin and preterm infants and the optimal leptin intake. A study showed that adiponectin might affect short-term growth, but this was not seen with leptin [59]. Other results revealed that both hormones affected physical growth outcomes in preterm infants [60]. Furthermore, animal studies have demonstrated that leptin deficiency is associated with structural and functional brain alteration and lower brain weight [24,25,61]. Preterm infants are at high risk for leptin deficiency after birth [19,23], and the low leptin concentration in pasteurized human breast milk might have an additional effect on brain maturation and development. More research is needed, especially long-term studies on neurodevelopment. The risk of perinatal leptin deficiency is increased by breastfeeding, preterm birth, intrauterine growth restriction, and in male infants [62,63,64]. The leptin contents did not differ significantly between preterm and term milk, which is consistent with results in the literature [65]. This study highlights that unpasteurized HM is of major importance for preterm infants’ nutrients to avoid possible negative effects on brain maturation and development in this special group of vulnerable infants.

Although the effects of gestational age on HM composition are conflicting [8,66,67,68,69,70], our study indicates that GA has an impact on the nutritional content of HM. Mothers of preterm infants provided HM with significantly lower protein levels compared to lactating mothers of term infants. However, in interpreting these results, the following factors have to be mentioned in detail. Our group of term infants was relatively small compared to the preterm infant groups. Moreover, the group of term infants showed a longer lactation period, and it is well known that protein contents in HM decrease over the lactation period [8,69,71].

A strength of this study was the sample size compared to previous studies [46,51,52,72]. The collection, processing, and analysis of the HM were standardized in order to ensure comparability across all samples. This study was overall very well controlled, but the lower sample size in the term group has to be mentioned, and the measurements of the adipose tissue markers were not feasible in 15 HM samples.

In summary, we agree with Gidrewicz and Fenton, who reported that GA is an important predictor of HM composition [8]. Hence, GA should definitely be considered in the nutritional management of preterm infants to optimize their enteral daily protein intake and to prevent any protein malnutrition, which might result in inappropriate growth and development, especially in preterm infants.

## 5. Conclusions

In conclusion, our results show that HM processing had a significant impact on the leptin concentrations but had no effect on the protein levels. These data support the use of unpasteurized human milk for preterm infants’ nutrients to avoid possible negative effects on brain development. The milk of mothers from preterm compared to full-term infants differed, underlining the importance of individualized target fortification, especially focusing on protein.

## Figures and Tables

**Table 1:** Demographic characteristics of mothers and infants.

Parameter	*n* = 136
Maternal parameters	
Age of mother (years)	32 ± 7
Height (cm)	167 ± 6
Weight (kg)	68 ± 10
BMI (kg/m^2^)	24.5 ± 3.5
Primiparous, *n* (%)	77 (56.6)
Cesarean delivery, *n* (%)	124 (91.2)
Lactation period (days)	41 ± 32
Frequency of breastfeeding/pumping per day	6 ± 2
Infant parameters	
Male sex, *n* (%)	76 (55.9)
Gestational age (wk + d)	30 + 3 (± 5 + ± 2)
Preterm infants <37 weeks of GA, *n* (%)	126 (92.65)
Birth weight (g)	1525 ± 907
Birth height (cm)	38.8 ± 7.1
Head circumference (cm)	27.6 ± 4.5

Categorical data are presented as numbers (with percentages). Continuous data are presented as the mean ± standard deviation. BMI, body mass index; wk, week; d, day; GA, gestational age.

**Table 2:** Human milk nutrient compositions in different processing types.

	Fresh HM (*n* = 136)	Fresh Pasteurized HM (*n* = 136)	Thawed HM (*n* = 136)	Thawed PasteurizedHM (*n* = 136)
True protein (g/100 mL)	1.14 ± 0.38	1.11 ± 0.39	1.12 ± 0.39	1.09 ± 0.39
Crude protein (g/100 mL)	1.39 ± 0.47	1.37 ± 0.48	1.39 ± 0.48	1.36 ± 0.47
Fat (g/100 mL)	2.96 ± 0.87	2.87 ± 0.80	2.82 ± 0.82	2.83 ± 0.80
Carbohydrates (g/100 mL)	7.48 ± 0.69	7.48 ± 0.66	7.48 ± 0.73	7.44 ± 0.68
Energy (kcal/100 mL)	63.19 ± 9.80	62.46 ± 8.86	61.96 ± 9.53	61.79 ± 9.33
Total solids (g/100 mL)	12.15 ± 1.30	12.06 ± 1.19	11.99 ± 1.35	11.92 ± 1.33
Adiponectin (ng/mL)	22.10 ± 17.37	20.20 ± 12.56	21.28 ± 16.49	19.84 ± 11.41
Leptin (ng/mL)	0.19 ± 0.24	0.02 ± 0.05	0.14 ± 0.21	0.02 ± 0.06

Results are presented as means ± SD. HM, human milk; SD, standard deviation.

**Table 3:** Maternal and infant characteristics divided into different GAs.

	Extremely Preterm (*n* = 62, 46%)	Very Preterm (*n* = 30, 22%)	Moderate/Late Preterm (*n* = 34, 25%)	Term Infant (*n* = 10, 7%)
Maternal age (years)	31 ± 7	32 ± 7	35 ± 7	33 ± 5
BMI (kg/m^2^)	24 ± 3.5	24.5 ± 2.7	24.7 ± 3.5	26.9 ± 4.5
Lactation period (days)	33 ± 31	39 ± 30	53 ± 33	57 ± 31
Breastfeeding/pumping per day	5 ± 2	5 ± 2	6 ± 2	7 ± 1
Male sex, *n* (%)	35 (56%)	17 (57%)	21 (62%)	3 (30%)
Cesarean delivery, *n* (%)	61 (98%)	29 (97%)	29 (85%)	5 (50%)
Gestational age (wk + d)	26 + 1 (± 3 + ± 2)	29 + 1 (± 4 + ± 2)	34 + 1 (± 3 + ± 2)	39 + 1 (± 3 + ± 2)
Birth weight (g)	863 ± 192	1421 ± 821	2250 ±348	3472 ± 478
Birth height (cm)	33.1 ± 2.8	38.1 ± 5.3	45.7 ± 1.7	51.8 ± 2.8
Head circumference (cm)	24.5 ± 2.4	26.7 ± 3.4	31.6 ± 1.5	36.1 ± 2.6

Categorical data are presented as numbers (with percentages). Continuous data are presented as the mean ± standard deviation. BMI, body mass index; wk, week; d, day; GA, gestational age.

**Table 4:** Human milk nutrient composition in different GAs.

	Extremely Preterm HM (*n* = 62, 46%)	Very Preterm HM (*n* = 30, 22%)	Moderate/Late Preterm HM (*n* = 34, 25%)	Term Infant HM (*n* = 10, 7%)
True protein (g/100 mL)	1.03 ± 0.37	1.12 ± 0.33	1.29 ± 0.39	1.34 ± 0.34
Crude protein (g/100 mL)	1.26 ± 0.45	1.36 ± 0.39	1.59 ± 0.49	1.63 ± 0.42
Fat (g/100 mL)	2.90 ± 0.91	2.81 ± 0.91	3.25 ± 0.80	2.78 ± 0.59
Carbohydrates (g/100 mL)	7.52 ± 0.65	7.20 ± 0.75	7.62 ± 0.66	7.63 ± 0.79
Energy (kcal/100 mL)	62.24 ± 10.26	61.23 ± 9.20	66.81 ± 9.62	62.70 ± 6.88
Total solids (g/100 mL)	12.07 ± 1.31	11.74 ± 1.31	12.69 ± 1.23	12.10 ± 1.13
Adiponectin (ng/mL)	21.92 ± 20.36	19.51 ± 6.12	24.33 ± 19.31	23.13 ± 13.95
Leptin (ng/mL)	0.18 ± 0.28	0.21 ± 0.17	0.20 ± 0.25	0.17 ± 0.18

Results are presented as means ± SD. Categorical data are presented as numbers (%). HM, human milk; SD, standard deviation.

## Data Availability

The data presented in this study are available upon request from the corresponding author.
